# A Study of One-Class Classification Algorithms for Wearable Fall Sensors

**DOI:** 10.3390/bios11080284

**Published:** 2021-08-19

**Authors:** José Antonio Santoyo-Ramón, Eduardo Casilari, José Manuel Cano-García

**Affiliations:** 1 Departamento de Tecnología Electrónica, Universidad de Málaga, 29071 Málaga, Spain; jasantoyo@uma.es; 2 Departamento de Tecnología Electrónica, Universidad de Málaga, Instituto TELMA, 29071 Málaga, Spain; jcgarcia@uma.es

**Keywords:** fall detection system, inertial sensors, accelerometers, dataset, machine learning, one-class classifiers

## Abstract

In recent years, the popularity of wearable devices has fostered the investigation of automatic fall detection systems based on the analysis of the signals captured by transportable inertial sensors. Due to the complexity and variety of human movements, the detection algorithms that offer the best performance when discriminating falls from conventional Activities of Daily Living (ADLs) are those built on machine learning and deep learning mechanisms. In this regard, supervised machine learning binary classification methods have been massively employed by the related literature. However, the learning phase of these algorithms requires mobility patterns caused by falls, which are very difficult to obtain in realistic application scenarios. An interesting alternative is offered by One-Class Classifiers (OCCs), which can be exclusively trained and configured with movement traces of a single type (ADLs). In this paper, a systematic study of the performance of various typical OCCs (for diverse sets of input features and hyperparameters) is performed when applied to nine public repositories of falls and ADLs. The results show the potentials of these classifiers, which are capable of achieving performance metrics very similar to those of supervised algorithms (with values for the specificity and the sensitivity higher than 95%). However, the study warns of the need to have a wide variety of types of ADLs when training OCCs, since activities with a high degree of mobility can significantly increase the frequency of false alarms (ADLs identified as falls) if not considered in the data subsets used for training.

## 1. Introduction

According to the World Health Organization (WHO), a fall is defined as an involuntary event that results in a person losing their balance and coming to lie unintentionally on the ground or other lower level [1]. Despite the fact that the majority of falls are not fatal, it is estimated that 646,000 fatal falls occur annually, which makes them the second worldwide cause of death due to accidental injuries [1].

Fall-related health problems are particularly serious among older people as they are strongly associated to loss of autonomy, impairment, and early death. In the world, about 28–35% of adults over 65 suffer one or more falls per year, while this percentage rises to 32–42% among those over 70 [2]. This situation poses a logistical and economic challenge for national health systems, especially if we think that the share of population aged over 60 will double in 2050, reaching a figure of 2 billion people, compared to 900 million in 2015 [3]. This problem is aggravated as a significant proportion of older adults live alone, so that if an accident occurs, a caregiver (a family member, medical or nursing staff, etc.) must be alerted to provide help. In this context, the time that elapses between a fall and the moment in which the person is assisted has been shown to determine the physical aftermaths of the accident and even the probability of survival [4]. Consequently, the last decade has witnessed an increasing interest in the development of affordable Fall Detection Systems (FDSs), which are able to permanently monitor patients and to trigger an automatic alarm message to a remote agent as soon as the occurrence of a fall is presumed.

Existing FDSs can be categorized into two generic groups. Firstly, context-aware systems are grounded on the deployment of cameras, microphones, and/or other environmental sensors in the specific locations where the user must be monitored. On the other hand, wearable-based systems utilize small transportable sensors that can be easily integrated or attached to the users’ clothing or garments to measure different parameters that describe their mobility.

When compared to context-aware solutions, the monitoring provided by wearable architectures offers a more ubiquitous service as they are not restricted to the particular area where the contextual sensors are installed. In addition, they are less privacy intrusive than camera-based methods and more robust to the presence of external artifacts or the alteration of the user’s setting. In addition, this type of FDS can benefit from the widespread acceptability and decreasing costs of wearable devices (smartwatches, sport bands, etc.).

The fundamental purpose of automatic fall detectors is to achieve the most accurate discernment between falls and other movements or Activities of Daily Living (ADLs), by simultaneously minimizing the number of undetected falls and false alarms (ADLs misjudged as falls). The efficiency of an FDS relies on the algorithm that makes the detection decision after processing and analyzing the measurements that are constantly captured by the wearable sensors (mainly, accelerometers, less frequently, gyroscopes, and in some prototypes, magnetometers, barometers, or heart rate sensors).

Detection strategies can be roughly classified into two groups [5]: threshold-based and machine learning methods. Threshold-based algorithms assume that a fall has occurred when one or several parameters (derived from the sensor measurements) exceeds or drops below a certain threshold limit. These algorithms are easy to implement and have a low computational load, although they are too simplistic and rigid to correctly classify many complex movements (especially those ADLs that involve an intense physical activity). Contrariwise, algorithms based on machine learning models usually overperform the thresholding schemes [6], as they have a greater potential to self-adapt to a wider typology of ADLs and falls, by directly learning from a set of samples or movement traces and without requiring the explicit and heuristic definition of a threshold value.

In most studies of the related literature, machine learning algorithms follow a fully supervised approach, so they need to be trained with labeled examples of both ADLs and falls. However, falls are rare events, and most studies on FDS are almost completely determined by the lack of real-world fall examples. Owing to the evident difficulties of capturing samples of actual falls experienced by the target public of these systems (older adults), falls aimed at training and testing new proposals on FDSs have to be normally generated in a testbed through the movements of young and healthy volunteers that emulate falls on cushioned surfaces according to a systematic and predefined test plan.

The validity of this procedure is still under discussion. Some related studies [7,8] have compared the dynamics of the falls experienced by older people and those ‘mimicked’ by young subjects in an experimental environment. Authors concluded that although there are similarities between the characteristics of both fall patterns, there also exist relevant differences in the monitored magnitudes related to the reaction time and the mechanisms of the compensatory movements to avoid falling or further damages. In this respect, Aziz et al. showed in [9] that the effectiveness of some supervised learning algorithms may dramatically decrease when they are evaluated in real scenarios.

To cope with this problem, one-class classifiers (OCCs) are a subtype of machine learning architectures particularly adequate to develop binary pattern classifiers with heavily unbalanced datasets [10]. OCCs bypass the need of obtaining laboratory samples of the minority class (falls), as they are conceived to be exclusively trained with traces of the most common class (ADLs). In this way, in the case of FDSs, once the training of the system is accomplished, a fall is detected whenever a certain movement is classified as a ‘anomaly’ (‘novelty’ or ‘outlier’). This occurs when its features substantially diverge from the samples of the majority class used during the training phase.

In a real use scenario, FDSs will have most likely to be adjusted or ‘tuned’ to the particular dynamics of the movements of the user to be monitored. In this vein, Medrano et al. evinced in [11] the benefits of ‘personalizing’ the configuration of the FDS by training the models with movements generated by the final user. Obviously, this process should not oblige the patient to emulate or generate fall patterns to particularize the FDS. In this regard, OCCs may greatly ease the implementation of this system personalization, as long as any user could train from scratch a certain machine learning method just by wearing the system during a certain training period in which the sensors could collect the traces generated by the daily routines of the user and feed the detector.

The idea of utilizing OCCs as the decision core of an FDS is not new. Table 1 summarizes the works that have assessed the performance of anomaly detectors when they are programmed to detect falls with a wearable device. In some specific cases, the FDS develops a ‘hybrid’ approach by combining an OCC and a thresholding method (such as the proposal by Viet et al. in [12]) or an OCC and a fully supervised classifier (such as that proposed by Lisowska et al. in [13]).

In all cases, the algorithms are primarily based on the analysis of the signals captured by a triaxial accelerometer, which is a strategy that has been massively adopted by the related literature on wearable FDSs. Only in six papers the information provided by the accelerometer is complemented by the use of other inertial sensors (a gyroscope, a magnetometer, or an orientation sensor), and in just two cases, a more complex sensor-fusion policy is applied, so that the classifiers are also fed with signals captured by other type of wearable sensing units (e.g., a heart rate monitor in the paper by Nho et al. [14]).

Table 1 indicates the best reported performing metrics (normally expressed in terms of sensitivity or specificity) of the corresponding OCC in the review literature. When more than one type of classifier is compared, the best performing algorithm in each study is marked in bold in the third column of the table. The results show that in some works, OCCs may achieve a noteworthy efficacy to discriminate ADLs from falls (with sensitivities and specificities higher than 0.98 or 98%). Furthermore, in [15], Medrano et al. illustrate that one-class classifiers may even exhibit a significantly better performance than their supervised counterparts. However, as it can be also appreciated from the last column in the table, all the works employ only one or at most two datasets to evaluate these algorithms. In some studies, these datasets are not obtained from a public repository but directly generated (and not released) by the authors. Due to the limited number of subjects and types of ADLs and falls considered in these datasets, it is legitimate to question if these results can be extrapolated to other repositories. Furthermore, the design criteria of these benchmarking datasets do not follow any particular recommendation and strongly rely on particular decisions of their creators. In a recent work [16], we have shown that even a deep learning method may achieve very divergent results when it is applied to different datasets. Thus, the good performance metrics obtained with a certain repository should be confirmed by training and testing the classifier with other datasets.

Another key problem of OCCs that is normally neglected by the related literature relates to the fact that these detectors may produce false alarms when tested with types of ADLs that were not part of the training subset [17]. This situation would be not so uncommon in a realistic scenario where the monitored user may execute unexpected movements (not caused by falls) that can be consequently be catalogued as ‘anomalies’ by the detector and trigger an undesired alerting message. Contrariwise, in the previous works on OCC-based FDSs, the ADLs included in the data subsets used for testing incorporate the same types of movements utilized for the configuration of the detector, which inherently minimizes the possibility of experiencing these false alarms.

In this paper, we thoroughly analyze these two issues. To this end, we systematically analyze the behavior of five basic types of anomaly detectors (with diverse hyperparameter configurations and input feature sets) when they are employed with nine different well-known datasets captured on the same body positions (the waist). We also investigate if the classification efficacy degrades when new of types of ADLs (not considered for training) are used for testing.

The paper is organized as follows: after the introduction and analysis of the related works presented in this section, Section 2 describes the different aspects of the methodology followed to evaluate the classifiers. Section 3 displays and discussed the main results for the considered study cases. Finally, Section 4 recapitulates the main conclusions of the article.

## 2. Methods

### 2.1. Election of the Datasets

To date, there have been released about 25 available datasets to benchmark detection algorithms for transportable FDSs (see [33] for a comprehensive review on this topic). These databases are formed by a set of numerical traces describing the signals captured by inertial sensors placed on one or several locations of the body. To the best of our knowledge, just one released dataset, provided by the FARSEEING project [34], publicly offers a very limited and unrepresentative number of traces captured from actual falls of older adults. In the other cases, the repositories are generated by recruiting a group of volunteers that systematically execute or emulate a series of predetermined ADLs or falls while transporting the corresponding sensor or sensors. For each movement, a trace (labeled as ADL or fall) is created.

Several studies [35,36,37,38,39] have shown that FDSs located on the waist outperform those placed on other body positions with a higher and independent mobility (e.g., a limb) as long as the waist is adjacent to the center of gravity of the human body. Therefore, in order to set up a common reference framework under optimal conditions, we limit our analysis to those 15 repositories that offer inertial data measured at the waist (although some of them also contain measurements captured on other body positions). For the study, we also discard those datasets that do not provide a significant number of samples (less than 400) or those that were collected with an accelerometer range of 2 g, which is too small to properly characterize the abrupt acceleration peaks caused by falls. After applying these criteria, we selected the 9 datasets (DLR, DOFDA, Erciyes, FallAllD, IMUFD, KFall, SisFall, UMAFall, and UP-Fall) described in Table 2. This quantity is clearly superior to the number of benchmarking repositories that are typically considered by the related literature to assess the performance of fall detection algorithms (in fact, as confirmed in Table 1, most proposals are validated against a single dataset). This need of evaluating the classifiers with different repositories is critical if we consider the remarkable heterogeneity [33,40] that exists among the available datasets in terms of the typology of the emulated ADLs and falls, strategies to generate the movements, duration of the traces, environment for the testbed, election of the volunteers, etc.

### 2.2. Compared One-Class Classifying Algorithms

As aforementioned, one-class classifiers constitute a particularization of binary supervised classification systems, in which the detection algorithms are trained only with data of one class. After the classifier is trained on these one-class traces, data corresponding to a category different from that used during training can be detected as anomalies. Therefore, once the model of an OCC is developed, input patterns can be identified as anomalies when a certain parameter derived from the input signals (e.g., a distance) exceeds a predefined decision threshold.

In the case of FDSs, the concept of an anomaly fits well with that of a fall, which can be envisaged as an unexpected movement that presents atypical characteristics with regard to those of the common or majority class (ADLs). Thus, in our evaluation, the classifiers are trained exclusively with part of the ADL samples included in the datasets while they are tested with both the falls and the rest of the ADLs (those not employed during the training stage).

In order to thoroughly evaluate the feasibility of using an OCC as the core of FDSs, we analyze the performance of five well-known one-class classifiers [10]: an autoencoder, a Gaussian Mixture Model (GMM), a Parzen Probabilistic Neural Network (PPNN), a One-Class K-Nearest Neighbor (OC-KNN), and One-Class Support Vector Machine (OC-SVM). All the classifiers were implemented and executed with Matlab scripts that used the Statistics and Machine Learning Toolbox [48]. Table 3 summarizes the values and possible considered alternatives to hyper-parameterize these classifiers. Through a grid search, we evaluated the performance of the algorithms for the different combinations of these hyperparameters.

As the decision threshold to detect the anomaly for each OCC, we employ the variable described in Table 4.

### 2.3. Feature Selection

In order to characterize the mobility samples and feed the machine learning classifiers, we compute a set of features derived from the raw signals collected by the inertial sensors. As all the repositories include the data captured by an accelerometer, which is the most employed sensor in the literature on wearable FDSs, the features are derived from the triaxial acceleration measurements. Falls provoke sudden peaks of the acceleration magnitude when the body hits the ground. This Signal Magnitude Vector (*SMV_i_*), for the *i*-th measurement, is computed as:

(1) $$SMV_{i}=\sqrt{A_{x_{i}}^{2}+A_{y_{i}}^{2}+A_{z_{i}}^{2}}$$

where $A_{x_{i}},{\;A}_{y_{i}},{\;\mathrm{and}\;A}_{z_{i}}$ indicate the values of triaxial components of the acceleration for each axis. For every movement trace (ADL or fall), the feature extraction exclusively focuses on a time interval of ±1 s around the sample where the maximum value of *SMV_i_* is identified, while the rest of the measurements in the sequence are not considered. The election of the duration of this observation window of 2 s (centered around the acceleration peak) is justified by the fact that an interval between 1 and 2 s is a good trade-off between recognition speed and accuracy to recognize most human activities [49]. In any case, the duration of the critical (impact) phase of a fall does not typically last longer than 0.5 s [50,51]. Thus, all the features will be derived from the consecutive acceleration components collected in the interval: $\left[i_{o}-N_{W},i_{o}+N_{W}\right]$ where *i_o_* is the index of the sample in which the maximum acceleration module is located:

(2) $$SMV_{i_{o}}=\max\left(SMV_{i}\right)\;\forall i\in \left[1,N-N_{W}+1\right])$$

where *N* denotes the number of measurements of in the trace (for each axis), while *N_W_* describes the number of samples captured during the observation window. *N_W_* can be straightforwardly calculated as:

(3) $$N_{W}=f_{s}\;\cdot \;t_{w}$$

where *f_s_* is the sampling rate of the trace and *t_w_* is the total duration of the window (2 s).

As a proper selection of the input features is a crucial factor in the design of any machine learning method, we consider different alternative candidate feature sets.

Firstly, we employ a set of twelve statistical candidate features that are physically interpretable as they entail a certain characterization of human dynamics. These features have been utilized by other works in the related literature on fall detection and activity recognition systems (refer, for example, to the comprehensive studies presented by Vallabh in [52] or by Xi in [53]). The symbol, labels (or labeling identifiers) and description of these twelve features are presented in Table 5 (a more detailed formal description of these parameters is provided in [33]).

In order to select the most convenient combination of input features from these 12 candidate statistics, we performed a preliminary analysis of the effectiveness of these statistics when they are applied to the aforementioned datasets to discriminate falls and ADLs with the classifiers. For all the studies, all the features were z-score normalized before training and testing. After implementing all the possible permutations of the statistics to feed the detectors, obtained results (not presented here for the sake of simplicity) revealed that the two combinations that yielded the best performance metric (sensitivity and specificity) in the classifiers were those using the seven features labeled as *B*, *C*, *D*, *F*, *G*, *I*, and *K* in Table 4 (‘*BCDFGIK*’ feature set) as well as the set that employed the 12 candidate features (‘*ABCDEFGHIJKL*’ feature set).

As the election of these input feature sets can still seem arbitrary, we also consider another set of features obtained from the *hctsa* (Highly Comparative Time-Series Analysis) Matlab software package [54]. This software is capable of extracting thousands of heterogeneous features from a time-series dataset to produce an optimized low-dimensional representation of the data.

In our case, a set (HCTSA feature set) of 12 features has been selected according to the following procedure:

- The SisFall [31] repository is selected as the baseline reference as it is considered one of the most complete in terms of types and quantity of movements and number and typology of subjects.
- The candidate features of the samples are obtained by using HCTSA.
- The performance resulting from the classification of the data is calculated by using each characteristic as input of a Support Vector Machine classifier with linear kernel and a *k*-fold analysis (with *k* = 10).
- The tool analyzed the correlation between the features that have led to the best results. Then, the application was programmed to divide these features into 12 different clusters, grouping those that are correlated into the same cluster. From each cluster, *hctsa* selected the most representative feature (the one closest to the center of the cluster).

### 2.4. Performance Metrics and Model Evaluation

For each combination of the hyperparameters, input feature set, and dataset, we trained an instance of the five contemplated OCCs with a certain number of ADLs and tested it with both ADLs and falls of the same repository. To assess the capability of the one-class classifiers to discriminate both categories, we employed two metrics universally employed in the evaluation of binary classifiers: the sensitivity (*Se*) or recall, which is defined as the ratio of falls in the test subset that are properly recognized, and specificity (*Sp*), which is defined as the proportion of test ADLs that are not misidentified as falls. Unlike other metrics (such as the accuracy or F1 score), sensitivity and specificity are not affected if the data classes are unbalanced in the datasets. Once the model is trained, the classifier is tested with 2500 possible values of the detection threshold (between a minimum and maximum that respectively guarantee the maximization of the sensitivity and specificity). Through the estimation of *Se* and *Sp* for each value of the discrimination threshold, we compute the receiver operating characteristic curve (ROC curve), which represents the evolution of *Se* (true positive rate) against 1-*Sp* (false positive rate). From the curve, we graphically calculate the AUC (Area Under the Curve) as a metric commonly used to characterize the overall performance of the binary classifiers. Additionally, as another global performance metric of the system, which describes the trade-off between an adequate recognition rate of falls (high sensitivity) and the absence of false alarms (high specificity), we also utilize the geometric mean of *Se* and *Sp* ($\sqrt{Se\cdot Sp}$) (together with the values of *Se* and *Sp*) in the point of the ROC where the maximum of this statistic is found. The election of this optimal cut-point in the ROC to select the corresponding decision threshold has been also proposed in works such as [55].

In order to minimize the impact of the election of the data used for training and testing the models, we evaluated the classifiers by means of a *k*-fold cross-validation [56,57]. For that purpose, the ADL traces of all datasets were split in five partitions (*k* = 5). Thus, for each combination of OCC, hyperparameters, input feature set, and dataset, the classifier is independently trained and tested five times. For each iteration, one of the five different partitions is reserved for the testing phase, while the rest of the ADLs and all the falls in the corresponding database are used to test the model. The performance metrics obtained with the test datasets for the five iterations (AUC, *Se*, and *Sp* for the threshold value that yields the highest value of $\sqrt{Se\cdot Sp}$) are averaged to characterize the performance of the classifier.

## 3. Results and Discussion

### 3.1. Study for the ‘Fair’ Case

As previously commented and indicated in Table 2, the datasets were generated by considering different predetermined types of ADLs and falls, which were executed by the experimental subjects. In our first analysis, we investigate the performance of the OCCs when the different typologies of ADLs are evenly (‘fairly’) distributed among the five subsets for five-fold cross-validation. Thus, we guarantee that all the types of ADL movements are represented in the subsets with which the anomaly detectors are trained.

The performance metrics obtained for the five algorithms and the nine datasets are presented in Table 6. Due to the high number of combinations that were evaluated, for each dataset and each type of OCC, the table only shows the combination of hyperparameters and input feature set (also indicated in the table) with which the highest value of the geometric mean of sensitivity and specificity ($\sqrt{Se\cdot Sp})$ was achieved. For each dataset, the row corresponding to the classifier with the best global metric is highlighted in bold. Aiming at giving an insight of the confidence interval of the measurements, together with the mean value of the global metric $\sqrt{Se\cdot Sp}$, the table also includes in the last column (preceded by the sign ±) the standard deviation of this parameter obtained for the five tests of the corresponding k-fold validation of the classifier. To ease the visualization of the comparison of the algorithms, the particular results of the AUC and $\sqrt{Se\cdot Sp}$ are summarized in Table 7 and Table 8, respectively. The highest values are also emphasized in bold.

From the results, we can draw the following conclusions:

- The best results are achieved by the OC-KNN classifier, which outperforms the rest of the detection methods for five out of the nine analyzed datasets (in terms of the geometric mean of sensitivity and specificity), while it presents the second or third best results for the other three datasets.
- The one-class SVM detector produces the best results for three datasets, while it offers the second-best behavior for five repositories. In any case, if we take into account the confidence interval that can be derived from the measurements, we can conclude that the differences in the behavior of OC-KNN and OC-SVM are not statistically significant.
- In most cases, the best performance is attained with the simplest input feature set (with the seven features labeled as *BCDFGIK* and described in Table 5): This suggests that if the features are conveniently selected, a parsimonious OCC architecture can be sufficient to produce efficient detection decisions.
- GMM, autoencoder and, specially, PPNN classifiers offer a more variable and erratic behavior as the quality of the classification strongly depends on the employed datasets. In several databases, the best achieved geometric mean of sensitivity and specificity is under 0.90.
- For all the datasets, the OC-KNN classifier yields at least a specificity and a sensitivity of 0.9. In most cases, these metrics are both higher than 0.95. These results are in line with most of the supervised (double-class) methods of machine learning that can be found in the related literature (see, for example, the surveys presented in [58,59,60,61,62,63]). This implies that if the decision threshold is properly chosen, an OCC can behave as a two-class classifier without requiring training the detector with falls. In a realistic use scenario, the final user of the detector e.g., an older adult) could be monitored during his/her daily routines to generate a dataset of ADLs. This dataset could be used to train and personalize an FDS based on an OCC.

### 3.2. Study of the Benefits of Ensemble Learning

Ensemble methods offer a simple and efficient paradigm to boost the prediction capability of single machine learning methods basing on the combined decision of multiple models [64]. In this subsection, we assess if the aggregate knowledge reached by the models evaluated in the previous analysis can improve the individual performance of the classifiers. In particular, we re-calculate the detection decision when a simplistic majority voting of three classifiers is applied (a similar performance is achieved if a higher number of models is considered). In this case, for each dataset, we use as base learners the three combinations of hyperparameters, input feature sets, and OCCs with which the three highest global performance metrics (geometric mean of *Se* and *Sp*) were obtained. Thus, during the testing phase, a trace is identified as a fall if a majority of the decision classifiers (two or three) classify the movement as a fall.

The obtained results are presented in Table 9. For comparison purposes, the table also indicates the best results (extracted from Table 6) corresponding to the best discrimination ratio achieved by a single OCC. In the table, the metrics of the ensemble classifier are marked in bold when they improve those generated by the single learner. Conversely, the results are highlighted in italics when the majority voting underperforms the best single model.

As it can be observed, the use of the ensemble improves the global performance metric in six out of the nine analyzed datasets (in several cases, a value of $\sqrt{Se\cdot Sp}$ close to 0.99 is attained), while with just one repository (DLR), the application of the voting technique reduces the effectiveness of the binary classification process.

### 3.3. Impact of the Typology of ADLs Employed in the Training Phase

As mentioned above, OCCs avoid the need of obtaining (or generating) traces describing real or emulated falls that are required to train supervised learning algorithms. In contrast, the use of one-class classifiers can present difficulties related to a lower specificity of the system due to the appearance of a greater number of false alarms or false positives, which is caused by ADLs not contemplated in the training dataset and identified as anomalies.

To determine the extent of this problem, we repeat the previous study of Section 3.1 when a certain typology of ADLs is removed from the training set and included in the testing subset. For this purpose, as already suggested in our previous studies in [33,40], the ADL movements of all the repositories have been split into three categories, which are displayed in Table 10, depending on the physical effort that they required to perform.

For each dataset (except for the DOFDA repository, which does not include sufficient traces of two categories), we generated three subsets of ADLs containing the traces of the corresponding categories. The best combination of hyperparameters and input feature set of each type of OCC obtained in Section 3.1 is trained and tested three times. In each experiment, each model is exclusively trained with the subsets of two categories and then tested with the falls and ADLs of the remaining category using the optimal decision threshold computed for the ‘fair’ case.

The results for all the analyzed datasets and the best performing OCC of each type are shown in Table 11, Table 12 and Table 13 for the cases in which the training sets do not include basic, standard, and sporting activities, respectively. The last column of each table (‘Loss’) indicates the difference between the global performance metric obtained with this segregation of the training and test subsets based on the categorization of the ADLs and the performance metric achieved with the ‘fair’ case (Table 6) in which traces of all the categories of ADLs are incorporated into the training subset. Consequently, a negative value of this parameter denotes a deterioration of the recognition capacity of the classifier.

As it could be expected, results show that the presence of new types of ADLs in the testing sets (not considered during the training phase) causes a strong degradation of the capability of the classifiers to discriminate falls from ADLs. This loss of effectiveness is particularly remarkable in those repositories (such as FallAllD) that encompass a greater number of types of ADL.

In this regard, the poorest discrimination rate is achieved when the system is tested with sporting movements. In some datasets, the best results for this situation achieve specificities below 80% (which imply that more than 20% of sporting actions are considered as falls and would trigger a false alarm). The brusque mobility patterns induced by this category of movements obviously provoke that the classifiers (trained with much less agitated activities) misinterpret them as anomalies.

Paradoxically, the results also indicate that very basic and less energetic activities also result in false positives, as they can be also identified as ‘novelties’ if traces corresponding to low motion movements are not included in the training subset. Nevertheless, these false alarms originated by ‘sedentary’ actions could be most probably avoided by a simple thresholding technique so that a movement trace is inputted to the OCC only if the magnitude of the acceleration exceeds a certain value and a fall can be reasonably suspected.

Finally, the movements included in the standard category seem to be the typology of activities with the lowest impact on the effectiveness of the training. This can be explained by the fact that these activities represent an intermediate point of physical strength between basic and sporting movements. Thus, training with movements with a lower and greater intensity (basic activities and sports, respectively) gives enough information to the classifiers to avoid being considered as anomalies. Yet, a relevant decay of the performance of certain OCCs is also perceived when this category is excluded from the training phase.

## 4. Conclusions

This work has assessed the effectiveness of utilizing one-class classifiers as the decision core of fall detection systems based on wearable inertial sensors. Unlike fully supervised methods, OCCs benefit from the fact that they can be trained exclusively with samples of a single class (conventional Activities of Daily Living), which avoids the need of obtaining traces captured during falls to train the classifiers.

In particular, we have analyzed the performance of five well-known OCCs under different input feature sets and a wide selection of hyperparameters. In contrast with most studies in the literature, which base their analysis on the use of a single dataset, we have extended the study to nine public repositories.

The achieved results (with values of the geometric mean of sensitivity and specificity higher than 95%) have shown the capability of the OCC to discriminate falls from ADLs with a high accuracy if the election of the decision threshold is optimized. This performance is comparable to that obtained with supervised systems in the literature. For almost all tests and datasets, the one-class KNN classifier stood out as the best (or second best) detection algorithm, which is a conclusion that is coherent with other previous analysis in the related works. The study has also revealed that the use of simplistic ensemble learning methods (such as voting) may improve the hit rate of the detector if the decisions of several OCCs are simultaneously considered.

In any case, the analyses have illustrated the extreme vulnerability of these classifiers to the typology of the ADLs used for the training phase. Actions that involve rapid movements (such as sports) and even very basic activities (which do not require any physical effort) may be straightforwardly identified as anomalies if they are not considered in the patterns used for training. This problem, which could be alleviated with the combination of OCCs and other simple methods that avoid identifying certain typical ADLs as falls, forces rethinking the way in which one-class detectors are adjusted and evaluated. The results clearly show the importance of having a sufficiently varied set of samples for training. Likewise, in the test phase, and as stress tests of the system, the evaluation should ponder the use of ADLs (not used for training) that entail agitated movements that may affect the decision of the classifier. Future studies should also focus on methodologies that automatically optimize the election of the decision threshold.

## Figures and Tables

**Table 1:** Works that have proposed and compared one-class classifiers to detect falls as anomalies.

Ref. and Authors	Year	Type of Compared OCCs	Number of Features	Employed Sensors	Best Achieved Performance	Employed Datasets (Number of ADL/Falls)
**Zhang et al.** [18,19]	2006	**OC-SVM + KFD +k-NN,** OC-SVM	6	Acc	Se = 0.9703 Sp = 0.9521	-Unpublished dataset (676/418)
**Yin et al.** [20] ^2^	2008	**OC-SVM + KNLR,** OC-SVM + MLLR, OC-SVM	n.i.	Light, Temp., Mic., Acc., Mag.	AUC = 0.985 Se = 0.90 Sp = 0.93	-Unpublished dataset (431/112 near falls)
**Viet and Choi** [12]	2011	**1-SVM**	4	Acc, Ori,	Se = 0.7699	-Unpublished dataset (n.i./226)
**Medrano et al.** [21]	2014	OC-KNN, OC-SVM, OC-KNN-sum, **Kmeans + OC-KNN**	51	Acc	AUC = 0.957 Se = 0.929 Sp = 0.890	-tFall [21] (9883/1026)
**Khan et al.** [22,23]	2014 2017	**XHMM**, HMM, OC-KNN, OC-SVM	31	Acc, Gyr.	Se = 0.893 Sp = 0.970	-DLR [24] (961/56) -MobiFall [25] (342/288)
**Lisowska et al.** [13]	2015	RNN, OC-SVM, **OC-KNN**	21	Acc	Se = 0.858 Sp = 0.853 AUC = 0.915	-Unpublished dataset (641/168)
Medrano et al. [11]	2016	**OC-KNN**, OC-SVM, LOF	153	Acc	AUC = 0.9809 Se = 0.9541 Sp = 0.9484	-tFall [21] (9883/1026)
Yang et al. [26] ^1^	2016	**OC-SVM**	38	Acc, Gyr.	Se = 0.783 Accuracy = 0.852	-Unpublished dataset (n.i./252 near falls)
Medrano et al. [15]	2017	**KDE**	4	Acc	Se = 0.986 Sp = 0.972	-tFall [21] (9883/1026)
Khan and Taati [27]	2017	**Ensemble of AEs,** OC-KNN, OC-SVM	6	Acc, Gyr	$\sqrt{Se\cdot Sp}$ = 0.959	-DLR [24] (961/56) -Cogent Labs [28] (1520/448)
Micucci et al. [29]	2017	**OC-KNN**, OC-SVM	12, 51, 384	Acc.	AUC = 0.997 Se = 0.996 Sp = 0.993	-tFall [21] (9883/1026) -HAR database [30] (360/0)
Lisowska et al. [31]	2018	RNN, OC-SVM, **OC-KNN**	21	Acc	AUC = 0.950	-Unpublished dataset (641/168) -tFall [21] (9883/1026)
Chen et al. [32]	2019	**Ensemble of AEs + OCCCH,** OCCCH, OC-SVM	3 windows of 500 samples	Acc.	Se = 0.9913, Sp = 0.9625	-Unpublished dataset (288/234)
Nho et al. [14]	2020	**GMM**	22	Acc, HR	Se = 0.9309, Sp = 0.8958	-Unpublished dataset (273/126)

^1^ The system is actually designed to detect “near-miss falls” (not falls). **^2^** The system is actually designed to generically detect abnormal activities (not only falls). n.i. Not indicated by the authors in the article. Acronyms for the OCCs: AE (Autoencoder), GMM( Gaussian Mixture Model), HMM (Hidden Markov Model), KDE (Kernel Density Estimation), KFD (Kernel Fisher Discriminant), Kmeans (K-means clustering), KNLR: Kernel Non-Linear Regression, LOF (Local Outlier Factor), MLLR (Maximum Likelihood Linear Regression), OC-KNN (One-Class K-Nearest Neighbors), OC-KNN-sum (One-Class K-Nearest Neighbors with sum of the distances), OC-SVM (One-Class Support Vector Machines), OCCCH (One-Class Classification based on the Convex Hull), RNN (Replicator Neural Network), XHMM (X-Factor’ Hidden Markov Model). Acronyms for the employed sensors: Acc. (Accelerometer), Gyr. (Gyroscope), HR (Heart Rate monitor), Mag. (Magnetometer), Mic. (Microphone), Ori. (Orientation angle sensor: pitch and roll), Temp. (Temperature). Acronyms for the metrics: AUC (Area Under the Curve), Se (Sensitivity), SP (Specificity).

**Table 2:** Basic data of the employed datasets.

Dataset	Number of Subjects (Females/Males)	Number of Types of ADLs/Falls	Number of Samples (ADLs/Falls)	Duration of the Samples (s)	Captured Signals in Each Sensing Point ^1^	Number and Positions of the Sensing Points	Sampling Rate (Hz)
DLR [24]	19 (8/11)	15/1	1017 (961/56)	[0.27–864.33]	3 (A, G, M)	1: Waist (belt)	100
DOFDA [41]	8 (2/6)	5/13	432 (120/312)	1.96–17.262	4 (A, G, O, M)	Waist	33
Erciyes Univ. [42]	17 (7/10)	16/20	3302 (1476/1826)	[8.36–37.76]	1 (A)	6: Chest, Head, Ankle, Thigh, Wrist, Waist	25
FallAllD [43]	15 (7/8)	44/35	6605 (4883/1722)^3^	20	4 (A, G, M, B)	3: Waist, Wrist, Chest (lanyard around the neck)	238 (A, G) 80 (M) 10 (B)
IMUFD [6]	10 (n.i.)	8/7	600 (390/210)	[15–20.01]	3 (A, G, M)	7: Chest, Head, Left ankle, Left thigh, Right ankle, Right thigh, Waist	128
KFall [44]	32 (0/32)	21/15	5075 (2729/2346)	[2.03–40.86]	A, G, O	1: Waist (Low back)	100
SisFall [45]	38 (19/19)	19/15	4505 (2707/1798)	[9.99–179.99] s	3 (A, A, G)	Waist	200
UMAFall [46]	19 (8/11)	12/3	746 (538/208)	15 s (all samples)	3 (A, G, M)	5: Ankle, Chest, Thigh, Waist, Wrist	100 (Thigh) 20 (Rest)
UP-Fall [47]	17 (8/9)	6/5	559 (304/255)	[9.409–59.979]	2 (A, G)	5: Ankle, Neck, Thigh (pocket), Waist, Wrist	Around 18 Hz

^1^ A: Accelerometer, G: Gyroscope, M: Magnetometer, O: Orientation sensor.

**Table 3:** Values and alternatives of the hyperparameters utilized for the evaluated models of the classifiers.

One-Class Classifier	Hyperparameter	Value/Alternatives
**Autoencoder**	Number of hidden neurons	6, 10, 12, 15
	Encoder/decoder transfer function	Logistic Sigmoid $f\left(z\right)=\frac{1}{1+e^{-z}}$
	Number of epochs	1000
	Loss function	Mean squared error plus L2&sparsity regularization
	Sparsity regularization coefficient	1
	L2 weight regularization coefficient	0.001
**Gaussian Mixture Model (GMM)**	Type of covariance matrix	Diagonal
	Number of components	3, 5 and 7
**Parzen Probabilistic Neural Networks (PPNN)**	Window function	$f\left(x\right)=e^{x-1}$
**One-Class K-Nearest Neighbors (OC-KNN)**	Distance function	Euclidean, Minkowski, Chebychev, Cosine
	Number of neighbors	5, 10, 50
**One-Class Support Vector Machine (OC-SVM)**	Kernel functions	Linear, cubic, quadratic, medium gaussian

**Table 4:** Decision thresholds employed to detect anomalies for the five considered OCCs.

OCC	Description of the threshold
**Autoencoder**	MSE (Mean Square Error) between input and output
**GMM**	Negative log-likelihood of the Gaussian mixture model given the data input
**PPNN**	Score indicating the likelihood that a label comes from the training class
**OC-KNN**	Distance between the observation and the *k* closest neighbor
**OC-SVM**	Score indicating the likelihood that a label comes from the training class

**Table 5:** Values and alternatives of statistics analyzed to select the input feature set of the classifiers.

ID	Symbol	Description
*A*	$\mu_{SMV}$	The mean Signal Magnitude Vector (SMV)
*B*	$A_{\omega_{diff\left(max\right)}}$	Magnitude of the maximum variation of the acceleration components
*C*	$\sigma_{SMV}$	The standard deviation of SMV
*D*	$\mu_{\theta }$	The mean rotation angle
*E*	$\mu_{SMV_{\left(diff\right)}}$	The mean absolute difference between two consecutive samples of the acceleration module
*F*	$\mu_{Ap}$	Mean of the acceleration components that are parallel to the floor plane
*G*	$SMV_{Max}$	The peak or maximum of the SMV to describe the violence of the impact against the floor
*H*	$SMV_{Min}$	The “valley” or minimum of the SMV to characterize the phase of free-fall
*I*	$\gamma_{SMV}$	The skewness of SMV, which describes the symmetry of the distribution of the acceleration
*J*	SMA	The Signal Magnitude Area
*K*	E	Sum of the energy estimated in the three axes during the observation interval
*L*	$\mu_{R}$	Mean of the autocorrelation function of the acceleration magnitude captured during the observation interval

**Table 6:** Performance metrics (AUC, Se, Sp, and $\sqrt{Se\cdot Sp})$ for the best combination of hyperparameters of the classifiers when they are applied to the datasets under study.

Dataset	Features	OCC	Kernel/Distance	Neighbors/ Hidden Neurons/ Components	AUC	*Se*	*Sp*	$\sqrt{Se\cdot Sp}$ (µ ± σ)
**DLR**	BCDFGIK	Autoencoder	Logistic sigmoid	10	0.8976	0.9333	0.8748	0.9007 ± 0.0739
	**HCTSA**	**GMM**	**Diagonal**	**7**	**0.9483**	**1.0000**	**0.8784**	**0.9371 ± 0.0204**
	HCTSA	OC-KNN	Cosine	50	0.9460	0.9333	0.9298	0.9286 ± 0.0760
	ABCDEFGHIJKL	PPNN	-	-	0.5564	0.6667	0.5826	0.6165 ± 0.1283
	HCTSA	OC-SVM	Medium Gaussian	-	0.9068	0.9333	0.8481	0.8864 ± 0.0696
**DOFDA**	BCDFGIK	Autoencoder	Logistic sigmoid	12	0.9704	0.9638	0.9833	0.9733 ± 0.0201
	BCDFGIK	GMM	Diagonal	5	0.9762	0.9835	0.9833	0.9833 ± 0.0198
	BCDFGIK	OC-KNN	Euclidean	5	0.9727	0.9604	0.9833	0.9716 ± 0.0247
	BCDFGIK	PPNN	-	-	0.9934	0.9506	1.0000	0.9749 ± 0.0122
	**HCTSA**	**OC-SVM**	**Linear**	**-**	**0.9775**	**0.9803**	**1.0000**	**0.9901 ± 0.0930**
**Erciyes**	ABCDEFGHIJKL	Autoencoder	Logistic sigmoid	15	0.9795	0.9544	0.9436	0.9488 ± 0.0091
	BCDFGIK	GMM	Diagonal	3	0.9857	0.9648	0.9436	0.9541 ± 0.0106
	**BCDFGIK**	**OC-KNN**	**Cosine**	**5**	**0.9951**	**0.9846**	**0.9782**	**0.9814 ± 0.0028**
	ABCDEFGHIJKL	PPNN	-	-	0.9898	0.9616	0.9640	0.9627 ± 0.0061
	ABCDEFGHIJKL	OC-SVM	Cubic	-	0.9867	0.9654	0.9837	0.9745 ± 0.0042
**FallAllD**	BCDFGIK	Autoencoder	Logistic sigmoid	6	0.9070	0.8753	0.8159	0.8449 ± 0.0161
	BCDFGIK	GMM	Diagonal	7	0.9359	0.8581	0.8649	0.8613 ± 0.0149
	**BCDFGIK**	**OC-KNN**	**Cosine**	**10**	**0.9649**	**0.9290**	**0.9062**	**0.9175 ± 0.0162**
	ABCDEFGHIJKL	PPNN	-	-	0.8281	0.7699	0.7897	0.7793 ± 0.0196
	BCDFGIK	OC-SVM	Linear	-	0.9552	0.8903	0.9164	0.9029 ± 0.0227
**IMUFD**	ABCDEFGHIJKL	Autoencoder	Logistic sigmoid	15	0.9111	0.8238	0.8610	0.8419 ± 0.0269
	BCDFGIK	GMM	Diagonal	3	0.9491	0.8991	0.9159	0.9069 ± 0.0294
	**BCDFGIK**	**OC-KNN**	**Cosine**	**5**	**0.9710**	**0.9712**	**0.9212**	**0.9458 ± 0.0184**
	BCDFGIK	PPNN	-	-	0.9269	0.8227	0.9028	0.8608 ± 0.0294
	BCDFGIK	OC-SVM	Linear	-	0.9745	0.9668	0.9135	0.9393 ± 0.0109
**KFall**	ABCDEFGHIJKL	Autoencoder	Logistic sigmoid	12	0.9931	0.9727	0.9699	0.9713 ± 0.0059
	BCDFGIK	GMM	Diagonal	7	0.9875	0.9697	0.9506	0.9601 ± 0.0063
	**BCDFGIK**	**OC-KNN**	**Minkowski**	**5**	**0.9976**	**0.9893**	**0.9895**	**0.9894 ± 0.0026**
	HCTSA	PPNN	-	-	0.9906	0.9607	0.9450	0.9528 ± 0.0077
	ABCDEFGHIJKL	OC-SVM	Linear	-	0.9940	0.9705	0.9672	0.9688 ± 0.0098
**SisFall**	BCDFGIK	Autoencoder	Logistic sigmoid	6	0.9611	0.9304	0.8948	0.9124 ± 0.0054
	BCDFGIK	GMM	Diagonal	7	0.9483	0.9221	0.8418	0.8808 ± 0.0135
	BCDFGIK	OC-KNN	Cosine	5	0.9895	0.9521	0.9634	0.9578 ± 0.0061
	**HCTSA**	**PPNN**	**-**	**-**	**0.9898**	**0.9638**	**0.9575**	**0.9606 ± 0.0045**
	HCTSA	OC-SVM	Linear	-	0.9932	0.9583	0.9567	0.9575 ± 0.0073
**UMAFall**	BCDFGIK	Autoencoder	Logistic sigmoid	15	0.9802	0.9946	0.9416	0.9677 ± 0.0123
	BCDFGIK	GMM	Diagonal	5	0.9769	0.9629	0.9086	0.9353 ± 0.0164
	**BCDFGIK**	**OC-KNN**	**Cosine**	**10**	**0.9881**	**0.9895**	**0.9670**	**0.9781 ± 0.0109**
	HCTSA	PPNN	-	-	0.9710	0.9095	0.9593	0.9337 ± 0.0119
	**BCDFGIK**	**OC-SVM**	**Cubic**	**-**	**0.9924**	**0.9895**	**0.9670**	**0.9781 ± 0.0144**
**UP-Fall**	BCDFGIK	Autoencoder	Logistic sigmoid	6	0.9674	1.0000	0.9249	0.9616 ± 0.0152
	BCDFGIK	GMM	Diagonal	3	0.9680	0.9755	0.9052	0.9394 ± 0.0300
	BCDFGIK	OC-KNN	Cosine	10	0.9888	0.9918	0.9685	0.9800 ± 0.0070
	ABCDEFGHIJKL	PPNN	-	-	0.9709	0.9837	0.9605	0.9720 ± 0.0248
	**BCDFGIK**	**OC-SVM**	**Cubic**	**-**	**0.9912**	**0.9918**	**0.9842**	**0.9880** ± 0.0110

**Table 7:** Obtained AUC (Area Under the Curve) of the ROC for the best combination of hyperparameters of the classifiers.

	Dataset								
Classifier	DLR	DOFDA	Erciyes	FallAllD	IMUFD	KFall	SisFall	UMAFall	UP-Fall
Autoencoder	0.8976	0.9704	0.9795	0.9070	0.9111	0.9931	0.9611	0.9802	0.9674
GMM	**0.9483**	0.9762	0.9857	0.9359	0.9491	0.9875	0.9483	0.9769	0.9680
OC-KNN	0.9460	0.9727	**0.9951**	**0.9649**	**0.9710**	**0.9976**	0.9895	0.9881	0.9888
PPNN	0.5564	**0.9934**	0.9898	0.8281	0.9269	0.9906	0.9898	0.9710	0.9709
OC-SVM	0.9068	0.9775	0.9867	0.9552	0.9745	0.9940	**0.9932**	**0.9924**	**0.9912**

**Table 8:** Maximum obtained geometric mean of sensitivity and specificity ($\sqrt{Se\cdot Sp}$ ) for the best combination of hyperparameters of the classifiers.

	Dataset								
Classifier	DLR	DOFDA	Erciyes	FallAllD	IMUFD	KFall	SisFall	UMAFall	UP-Fall
Autoencoder	0.9007	0.9733	0.9488	0.8449	0.8449	0.9713	0.9124	0.9677	0.9616
GMM	**0.9371**	0.9833	0.9541	0.8613	0.9069	0.9601	0.8808	0.9353	0.9394
OC-KNN	0.9286	0.9716	**0.9814**	**0.9175**	**0.9458**	**0.9894**	0.9578	**0.9781**	0.9800
PPNN	0.6165	0.9749	0.9627	0.7793	0.8608	0.9528	**0.9606**	0.9337	0.9720
OC-SVM	0.8864	**0.9901**	0.9745	0.9029	0.9393	0.9688	0.9575	**0.9781**	**0.9880**

**Table 9:** Comparison of the performance metrics of the majority voting ensemble and those of the best single OCC.

Dataset	Algorithm	*Se*	*Sp*	$\sqrt{Se\cdot Sp}$
DLR	GMM (Diagonal. 7 components)	1.0000	0.8784	0.9371
	Majority Voting Ensemble	*0.9333*	**0.9146**	*0.9215*
DOFDA	OC-SVM (Linear kernel)	0.9803	1.0000	0.9901
	Majority Voting Ensemble	0.9803	1.0000	0.9901
Erciyes	OC-KNN (Cosine distance. 5 neighbors)	0.9846	0.9782	0.9814
	Majority Voting Ensemble	**0.9852**	0.9776	0.9814
FallAllD	OC-KNN (Cosine distance. 10 neighbors)	0.9290	0.9062	0.9175
	Majority Voting Ensemble	*0.9269*	**0.9223**	**0.9245**
IMUFD	OC-KNN (Cosine distance. 5 neighbors)	0.9712	0.9212	0.9458
	Majority Voting Ensemble	**0.9763**	**0.9342**	**0.9550**
KFall	OC-KNN (Minkowski distance. 5 neighbors)	0.9893	0.9895	0.9894
	Majority Voting Ensemble	**0.9987**	**0.9985**	**0.9986**
SisFall	OC-KNN (Cosine distance. 5 neighbors)	0.9638	0.9575	0.9606
	Majority Voting Ensemble	0.9638	**0.9627**	**0.9632**
UMAFall	OC-KNN (Cosine distance. 10 neighbors)	0.9895	0.9670	0.9781
	Majority Voting Ensemble	0.9895	**0.9771**	**0.9832**
UP-Fall	OC-SVM (Cubic kernel)	0.9918	0.9842	0.9880
	Majority Voting Ensemble	**0.9959**	*0.9841*	**0.9899**

**Table 10:** Categorization criteria to divide the ADL movements into different types.

ADL Category	Description	Examples
**Basic movements**	Simple movements of low intensity	Getting up of a bed or chair, sitting down, lying, turning over while lying down, standing, clapping hands, etc.
**Standard movements**	Routines of daily life that require intermediate physical effort or a certain degree of mobility	Walking at a normal pace, climbing up/down stairs, squatting, picking up an object from the floor, etc.
**Sporting movements**	Activities that require a higher physical effort and brusque and/or repetitive movements	Running, jogging, hopping, walking fast, etc.

**Table 11:** Results of the classifiers when they are tested with falls and basic activities and trained with the rest of ADL categories.

Dataset	OCC	*Se*	*Sp*	$\sqrt{Se\cdot Sp}$	Loss
**DLR**	Autoencoder	0.8125	0.4299	0.5910	−0.3097
	GMM	0.8125	0.3293	0.5172	−0.4199
	**OC-KNN**	**0.8125**	**0.7591**	**0.7854**	−0.1432
	PPNN	0.7500	0.0122	0.0959	−0.5206
	OC-SVM	0.4375	0.0427	0.1367	−0.7497
**Erciyes**	Autoencoder	0.9885	0.8457	0.9143	−0.0345
	GMM	0.9104	0.9213	0.9159	−0.0382
	**OC-KNN**	**0.9632**	**0.9074**	**0.9349**	−0.0465
	PPNN	0.9764	0.8164	0.8928	−0.0699
	OC-SVM	0.9863	0.8765	0.9298	−0.0447
**FallAllD**	Autoencoder	0.9555	0.7978	0.8639	+0.0190
	GMM	0.9140	0.8352	0.8737	+0.0124
	**OC-KNN**	**0.9269**	**0.8747**	**0.9004**	−0.0171
	PPNN	0.6688	0.7758	0.7203	−0.0568
	OC-SVM	0.9290	0.6813	0.7956	−0.1073
**IMUFD**	Autoencoder	0.9761	0.7387	0.8492	+0.0073
	GMM	0.9522	0.5495	0.7234	−0.1835
	**OC-KNN**	**0.9569**	**0.8378**	**0.8954**	−0.0504
	PPNN	0.8852	0.8108	0.8472	−0.0136
	OC-SVM	1.0000	0.7027	0.8383	−0.1010
**KFall**	Autoencoder	0.9944	0.8690	0.9296	−0.0417
	GMM	0.9851	0.8558	0.9181	−0.0420
	**OC-KNN**	**0.9624**	**0.9760**	**0.9692**	−0.0202
	PPNN	0.9018	0.9928	0.9462	+0.0005
	OC-SVM	0.9953	0.9183	0.9560	−0.0128
**SisFall**	**Autoencoder**	**0.9076**	**0.5626**	**0.7146**	−0.1978
	GMM	0.9950	0.3705	0.6072	−0.2736
	OC-KNN	0.9839	0.5073	0.7065	−0.2513
	PPNN	0.8520	0.0825	0.2650	−0.6956
	OC-SVM	0.9911	0.1574	0.3949	−0.5626
**UMAFall**	**Autoencoder**	**0.8670**	**0.8493**	**0.8581**	−0.1096
	GMM	0.9309	0.7226	0.8201	−0.1152
	OC-KNN	0.9894	0.7397	0.8555	−0.1226
	PPNN	0.9894	0.2226	0.4693	−0.4644
	OC-SVM	0.9947	0.6130	0.7809	−0.1972
**UP-Fall**	Autoencoder	0.9510	0.9881	0.9694	+0.0078
	GMM	0.9633	0.9881	0.9756	+0.0362
	**OC-KNN**	**0.9796**	**1.0000**	**0.9897**	+0.0097
	PPNN	0.9714	0.9762	0.9738	+0.0130
	OC-SVM	0.9918	1.0000	0.9959	+0.0079

**Table 12:** Results of the classifiers when they are tested with falls and standard movements and trained with the rest of ADL categories.

Dataset	OCC	*Se*	*Sp*	$\sqrt{Se\cdot Sp}$	Loss
DLR	Autoencoder	0.8125	0.9008	0.8555	−0.0452
	GMM	0.9375	0.8855	0.9111	−0.0260
	OC-KNN	0.8125	0.8168	0.8146	−0.1140
	PPNN	0.6875	0.0458	0.1775	−0.4390
	**OC-SVM**	**0.7500**	**0.9313**	**0.8357**	−0.0507
Erciyes	Autoencoder	0.9665	0.9817	0.9741	+0.0253
	GMM	0.9764	0.9854	0.9809	+0.0268
	OC-KNN	0.9780	0.9689	0.9735	−0.0079
	PPNN	0.9720	0.9689	0.9704	+0.0077
	**OC-SVM**	**0.9868**	**0.9634**	**0.9751**	+0.0006
FallAllD	Autoencoder	0.8258	0.8808	0.8528	+0.0079
	GMM	0.8602	0.8808	0.8704	+0.0091
	**OC-KNN**	**0.8903**	**0.9621**	**0.9255**	+0.0080
	PPNN	0.6774	0.7832	0.7284	−0.0487
	OC-SVM	0.8129	0.6667	0.7362	−0.1667
IMUFD	Autoencoder	0.8134	0.7833	0.7982	−0.0437
	GMM	0.9330	0.9667	0.9497	+0.0428
	**OC-KNN**	**0.9378**	**1.0000**	**0.9684**	+0.0226
	PPNN	0.7416	1.0000	0.8612	+0.0004
	OC-SVM	0.8565	1.0000	0.9255	−0.0138
KFall	Autoencoder	0.9983	0.9408	0.9691	−0.0022
	**GMM**	**0.9893**	**0.9723**	**0.9808**	+0.0207
	OC-KNN	0.9859	0.9781	0.9820	−0.0074
	PPNN	0.9061	0.9494	0.9275	−0.0182
	OC-SVM	0.9910	0.8177	0.9002	−0.0686
SisFall	Autoencoder	0.8620	0.6754	0.7630	−0.1494
	GMM	0.9182	0.5331	0.6996	−0.1812
	OC-KNN	0.9482	0.7756	0.8576	−0.1002
	PPNN	0.9727	0.7715	0.8663	−0.0943
	**OC-SVM**	**0.9488**	**0.8317**	**0.8883**	−0.0692
UMAFall	Autoencoder	0.9947	0.8936	0.9428	−0.0249
	GMM	0.9894	0.9362	0.9624	+0.0271
	OC-KNN	0.9787	0.9574	0.9680	−0.0101
	PPNN	0.8723	1.0000	0.9340	+0.0003
	**OC-SVM**	**0.9574**	**1.0000**	**0.9785**	+0.0004
UP-Fall	Autoencoder	0.9714	0.9675	0.9695	+0.0079
	GMM	1.0000	0.9431	0.9711	+0.0317
	**OC-KNN**	**1.0000**	**0.9512**	**0.9753**	−0.0047
	PPNN	1.0000	0.6423	0.8014	−0.1594
	OC-SVM	1.0000	0.6829	0.8264	−0.1616

**Table 13:** Results of the classifiers when they are tested with falls and sporting activities and trained with the rest of ADL categories (results for the IMUFD dataset are not included, as this repository does not include sporting movements).

Dataset	OCC	*Se*	*Sp*	$\sqrt{Se\cdot Sp}$	Loss
DLR	Autoencoder	0.9375	0.0294	0.1661	−0.7346
	GMM	0.9375	0.0294	0.1661	−0.7710
	OC-KNN	1.0000	0.0735	0.2712	−0.6574
	**PPNN**	**0.7500**	**0.8529**	**0.7998**	0.1833
	OC-SVM	0.3125	0.9706	0.5507	−0.3357
Erciyes	Autoencoder	0.5231	0.6848	0.5985	−0.3503
	GMM	0.9423	0.2609	0.4958	−0.4583
	OC-KNN	0.9758	0.9348	0.9551	−0.0263
	**PPNN**	**0.9500**	**0.9891**	**0.9694**	0.0067
	OC-SVM	0.9571	0.9457	0.9514	−0.0231
FallAllD	Autoencoder	0.6796	0.4176	0.5327	−0.3122
	GMM	0.7978	0.3235	0.5081	−0.3532
	**OC-KNN**	**0.9247**	**0.6294**	**0.7629**	−0.1546
	PPNN	0.8280	0.5235	0.6584	−0.1187
	OC-SVM	0.8731	0.6765	0.7685	−0.1344
KFall	Autoencoder	0.9377	0.4694	0.6635	−0.3078
	GMM	0.5137	0.6332	0.5703	−0.3898
	**OC-KNN**	**0.9684**	**0.7686**	**0.8627**	−0.1267
	PPNN	0.9667	0.7162	0.8320	−0.1137
	OC-SVM	0.9744	0.7358	0.8467	−0.1221
SisFall	Autoencoder	0.7607	0.7254	0.7428	−0.1696
	GMM	0.8564	0.3264	0.5287	−0.3521
	OC-KNN	0.9171	0.5181	0.6893	−0.2685
	**PPNN**	**0.9176**	**0.9793**	**0.9480**	−0.0126
	OC-SVM	0.9338	0.9378	0.9358	−0.0217
UMAFall	Autoencoder	0.9521	0.0000	0.0000	−0.9677
	GMM	0.9096	0.0000	0.0000	−0.9353
	OC-KNN	0.8989	0.5273	0.6885	−0.2896
	PPNN	0.8989	0.6182	0.7455	−0.1882
	**OC-SVM**	**0.7287**	**0.8545**	**0.7891**	−0.1890
UP-Fall	Autoencoder	0.8667	0.0000	0.0000	−0.9616
	GMM	0.7792	0.0000	0.0000	−0.9394
	**OC-KNN**	**0.9292**	**0.9565**	**0.9427**	−0.0373
	PPNN	0.9625	0.8913	0.9262	−0.0346
	OC-SVM	0.8898	0.9565	0.9226	−0.0654

## Data Availability

All the datasets employed in this work are publicly available. The URL to download the repositories can be found in the corresponding reference.
